# Effects of Three Different Doses of Dexmedetomidine and Ropivacaine on Analgesia and the Stress Response in Hypospadias Surgery: A Randomized Trial

**DOI:** 10.3389/fphar.2021.612216

**Published:** 2021-04-30

**Authors:** Yuan Wang, Ying-Ping Jia, Li-Yuan Zhao, Qiu-Juan He, Jin-Lian Qi, Rui Zhou, Ting Yang, Zeng-Xiao Zhao, Hao-Quan Wei

**Affiliations:** Department of Anesthesiology, HENAN Children’s Hospital, Zhengzhou Children’s Hospital, Zhengzhou University Children’s Hospital, Zhengzhou, China

**Keywords:** dexmedetomidine, anesthesia, caudal, children, hypospadias, comfort medical

## Abstract

**Objective**: This study was designed to investigate the effects of three different doses of dexmedetomidine in caudal blocks on postoperative stress and pain after pediatric urethroplasty.

**Methods:** A total of 160 children who underwent elective urethroplasty were enrolled in this study. They were randomly divided into four groups: groups D1, D2, and D3, in which the patients were injected respectively with a mixed solution of 1, 1.5, or 2 μg kg^−1^ of dexmedetomidine and 0.25% ropivacaine into the sacral canal; and group R, in which the patients were injected with 0.25% ropivacaine into the sacral canal. Cortisol and interleukin-6 (IL-6) levels within 24 h, the incidence of adverse events in the circulatory system during surgery, onset time of the caudal block, duration of postoperative analgesia, the incidence of agitation during recovery, and other anesthetic adverse reactions were observed and recorded.

**Results:** Compared with group R, cortisol and IL-6 levels in groups D1, D2, and D3 decreased within 24 h after the operation (T2–T6). The incidence of intraoperative hypertension, tachycardia, and shivering during the recovery period decreased, the onset time of the caudal block decreased, and the duration of postoperative analgesia increased (*p* < 0.01). Compared with group D1, the duration of postoperative analgesia increased in groups D2 and D3 (*p* < 0.01). Compared with groups D1 and D2, the incidence of excessive sedation and bradycardia in group D3 increased (*p* < 0.05).

**Conclusion:** The administration of 1.5 μg kg^−1^ of dexmedetomidine appears to be most feasible in accelerating the onset of the caudal block, reducing stress and inflammation, stabilizing the circulation, increasing the duration of postoperative analgesia, and reducing anesthesia- and operation-associated adverse events.

## Introduction

With the continuous improvements in the medical field, more attention is being paid to patient-centered care, and the special role of anesthesiology makes it an important focus for ensuring patient comfort (Li et al., 2018). Pediatric hypospadias surgery is very painful, leading to preoperative anxiety, perioperative stress response, postoperative agitation, pain, nausea, and even bleeding, infection, urethral fistula, and prolonged hospitalization (Wilson et al., 2017; Schröder et al., 2018). Used in neuraxial anesthesia, dexmedetomidine, an α2-adrenergic receptor agonist, can reduce the dosage of local anesthetics; reduce the onset time and prolong the block time (Shaikh and Mahesh, 2016; Xu et al., 2018); has sedative, analgesic, and stress-reducing effects; can be used for caudal blocks in children (Yang et al., 2015); and improves comfort and promotes rapid recovery in children. However, there are different reports about the appropriate dose (Lin et al., 2020; Zeng et al., 2020).

In this study, based on nasal sedation with dexmedetomidine, patients were given different doses of dexmedetomidine mixed with 0.25% ropivacaine and 0.25% ropivacaine alone. Perioperative stress and inflammatory response, the duration of postoperative analgesia, and anesthesia and surgical adverse events were compared to provide guidance for the comfortable medical care of children with hypospadias.

## Information and Methods

### General Information

The Ethics Committee of our hospital approved this study, and an informed consent form was signed by a family member of each of the patients.

A total of 160 children with type III hypospadias who underwent elective urethroplasty were enrolled in this study. Their age range was 3–6 years, their weight range was 13–26 kg, and the American Society of Anesthesiologists physical status classification was I or II. Patients were excluded from the study if they had a history of spinal disease or sacral canal closure, respiratory compromise or obstructive sleep apnea, congenital heart disease, epilepsy, communication or mental disorders, obesity (body mass index ≥18), local anesthetic allergy, severe liver and kidney dysfunction, genetic and metabolic diseases, or nasal mucosa injury (bleeding, scarring, or rupture of the nasal mucosa). The enrolled patients were divided into four groups (*n* = 40) according to the random number generator method: groups D1, D2, and D3, in which the patients were injected with a mixed solution of 1, 1.5, or 2 μg kg^−1^ of dexmedetomidine and 0.25% ropivacaine into the sacral canal; and group R, in which the patients were injected with 0.25% ropivacaine into the sacral canal.

### Preoperative Preparation

One day before the operation, the family members were given preoperative education, and the nurse anesthetist conducted psychological counseling for the children, who fasted from a solid diet for 6 h, from a liquid diet for 4 h, and from clear fluids for 2 h before the operation (a shorter-than-normal fasting time) (None. Practice, 2017).

One hour before the operation, the patient was taken to the waiting area, and perioperative information was provided in the form of animation on a large screen. The nurse anesthetist applied 5% compound lidocaine cream (1.5 g/10 cm2) to two venipuncture sites and one sacral puncture site and covered them with the infusion film. An intelligent robot communicated and interacted with children to reduce their tension and anxiety.

Midazolam (0.5 mg • kg^−1^) was given 20 min before the operation (configuration method: 10 mg • 2 ml^−1^ midazolam was diluted to 2.5 mg • ml^−1^ with 5% glucose injection. 0.2 ml • kg^−1^). The patient was placed in the Mecca position and dripped with 2 μg • kg^−1^ (0.02 ml • kg^−1^) of dexmedetomidine through bilateral nostrils. When the patient was anesthetized, they were taken into the operating room, and the vein was opened.

### Induction of Anesthesia

After entering the operating room, a vein was cannulated, the monitors were positioned, and baseline values were measured before anesthesia. Propofol (2–3 mg kg^−1^) and sufentanil (0.2–0.3 ug kg^−1^) were injected intravenously, and an LMA Supreme was inserted when the mandible was relaxed. The patient was connected to the Primus anesthesia machine (Dräger, Germany), and synchronized intermittent mandatory ventilation was adopted; the fraction of inspired oxygen (FiO_2_) was 40–60%, the flow rate was 3 L min^−1^, pulse oximetry levels (S_p_O_2_) was maintained at ≥95%, and end-respiratory partial pressure of carbon dioxide (P_et_CO_2_) was maintained within 35–45 mmHg (1 mmHg = 0.133 kPa). The patient was placed in the left lateral position. A 22-gauge trocar needle was inserted through the sacral hiatus into the sacral canal under ultrasound guidance using the M-Turbo ultrasound system (SonoSite Inc. Bothell, WA, United States). The injection dose was 1 ml kg^−1^ (maximum ≤20 ml) in each group. Radial artery catheterization was established, the pressure sensor was connected to measure arterial blood pressure, and a large vein was catheterized for blood sampling. The operation was started 15 min after the local anesthetic was injected.

### Anesthesia Maintenance and Recovery

During the operation, propofol (8–13 mg kg^−1^ h^−1^) was injected intravenously to maintain the bispectral index (BIS) within the range of 55–60 (Wang et al., 2019). After the operation, the medication was stopped, and the patient was sent to the recovery room. The laryngeal mask was removed when the frequency and amplitude of spontaneous respiration were satisfactory (VT > 8 ml kg^−1^, frequency >16 timesmin^−1^, P_ET_CO_2_ ≤ 45 mmHg).

### Monitoring and Observation of Indicators

BIS, mean arterial pressure (MAP), heart rate (HR), SpO_2,_ and P_ET_CO_2_ were monitored.

Stress and inflammation indicators—cortisol and interleukin-6 (IL-6): Before induction of anesthesia (T1), at the time of skin incision (T2), and at 4 h (T3), 8 h (T4), 12 h (T5), and 24 h (T6) after induction of anesthesia, 2 ml of venous blood was drawn, placed in an anticoagulant tube containing ethylenediaminetetraacetic acid, and centrifuged at 3,000 rpm for 10 min (the centrifuge radius was 12 cm). The serum obtained was preserved at −20°C. Radioimmunoassay was used to determine the concentrations of serum cortisol (the kit was purchased from Abbott, Chicago, United States) and IL-6 (the kit was purchased from Shanghai Ximei Biotechnology Co. Ltd. Shanghai, China).

Intraoperative cardiovascular adverse events: During the operation, bradycardia is defined as a 30% decrease from the baseline HR, and hypotension is defined as a 30% decrease from the baseline MAP. In these cases, 0.01 mg kg^−1^ of atropine and 0.5 mgkg^−1^ of ephedrine, respectively, were given intravenously. Additionally, tachycardia is defined as a 30% increase from the baseline HR, and hypertension is defined as a 30% increase from the baseline MAP. If either of these occurred, 0.1 μg kg^−1^ of sufentanil was given intravenously, and then the incidence was calculated and analyzed.

The onset time of the sacral canal block: the interval from the completion of the caudal block to the disappearance of the cremasteric reflex.

Duration of analgesia maintenance: The FLACC (face, legs, activity, cry, and consolability) pain assessment scale was used to evaluate the postoperative analgesic effect. A score ≥4 indicated that the child had pain. In this case, ibuprofen was given orally, and the time from the caudal block to oral medication was recorded.

Adverse effects of anesthesia: Postoperative nausea, vomiting, pruritus, excessive sedation (Ramsay score >4), respiratory depression, bradycardia, shivering, etc.

### Statistical Analysis

Data were statistically analyzed using SPSS19.0 statistical software (International Business Machines Corporation, Armonk, United States). The Shapiro–Wilk test was used to evaluate the normality of the data of each group, and *p* > 0.05 was considered normal distribution. Normally distributed measurement data were expressed as mean ± standard deviation $\left(\bar{\text{x}}\pm \text{SD}\right)$, and the inter-group comparison was conducted using univariate analysis of variance with the Bonferroni method. Count data were compared using a Chi-square test. *p* < 0.05 was considered statistically significant. A repeated-measures analysis of variance was also used to analyze the cortisol and IL-6 levels of each group at different time points, and a sphericity hypothesis test was performed with Mauchly’s test.

## Results

### General Information

The differences in general data and operation duration among all groups were not statistically significant (*p* > 0.05) (Table 1).

**TABLE 1:** General data of all patient characteristics ($\bar{\text{x}}\pm \text{SD}$, *n* = 40).

Group	Age (y)	Body weight (kg)	Operation duration (min)
R	4.3 ± 1.3	18.3 ± 4.6	128 ± 17
D1	4.5 ± 1.2	17.8 ± 4.7	131 ± 18
D2	4.2 ± 1.4	18.7 ± 4.8	129 ± 16
D3	4.4 ± 1.3	18.2 ± 4.7	130 ± 17

### Comparison of the Levels of Cortisol and IL-6 among the Four Groups

The results of the repeated-measures analysis of variance showed that the overall differences of cortisol and IL-6 levels between groups D1, D2, D3, and group R were statistically significant (*p* < 0.05). Interactions occurred between times and groups in cortisol and IL-6 levels (*p* < 0.05), that is, there were differences between groups D1, D2, D3, and group R at different time points (T2−T6).

Before induction of anesthesia (T1), there was no significant difference between the four groups. Compared with group R, cortisol and IL-6 levels in groups D1, D2, and D3 decreased from incision to 24 h after the operation (T2−T6) (*p* < 0.05), with the most significant decrease occurring between 8 and 12 h after the operation (T4−T5) (*p* < 0.01) (Tables 2,3).

**TABLE 2:** Stress response index of different timepoints ($\bar{\text{x}}\pm \text{SD}$, *n* = 40) (mol/L).

Group	R	D1	D2	D3
T1	393.54 ± 61.63	394.19 ± 60.28	393.46 ± 60.31	393.53 ± 69.37
T2	528.44 ± 62.24	504.38 ± 69.12^a^	501.76 ± 74.65^a^	502.29 ± 73.59^a^
T3	435.55 ± 63.33	411.77 ± 72.62^a^	408.74 ± 74.77^a^	409.38 ± 71.66^a^
T4	669.43 ± 70.37	393.62 ± 62.19^b^	386.73 ± 62.01^b^	397.72 ± 61.19^b^
T5	698.53 ± 62.83	435.65 ± 61.63^b^	410.57 ± 59.32^b^	429.44 ± 52.22^b^
T6	421.61 ± 65.77	399.43 ± 69.63^a^	397.25 ± 70.71^a^	398.38 ± 71.56^a^

(Stress response index was measured by the level of cortisol).

a *P* < 0.05 compared with Group R

b *P* < 0.01 compared with Group R

**TABLE 3:** Inflammatory response indexe of different timepoints ($\bar{\text{x}}\pm \text{SD}$, *n* = 40) (pg/ml).

Group	R	D1	D2	D3
T1	3.9 ± 1.6	3.9 ± 1.4	3.8 ± 1.5	3.8 ± 1.6
T2	7.2 ± 2.6	6.2 ± 2.8^a^	6.2 ± 2.9^a^	6.3 ± 2.7^a^
T3	7.3 ± 2.8	6.3 ± 2.9^a^	6.3 ± 2.8^a^	6.4 ± 2.7^a^
T4	28.2 ± 6.8	13.6 ± 4.1^b^	13.1 ± 4.2^b^	13.3 ± 4.6^b^
T5	24.3 ± 6.2	12.0 ± 4.7^b^	12.3 ± 4.1^b^	11.7 ± 4.9^b^
T6	10.2 ± 4.4	8.8 ± 4.1^a^	8.6 ± 4.4^a^	8.7 ± 4.5^a^

(Inflammatory response indexe was measured by the level of interleukin-6).

a *P* < 0.05 compared with Group R

b *P* < 0.01 compared with Group R

### Comparison of Anesthetic Effect and Adverse Events Among the Four Groups

Compared with group R, in groups D1, D2, and D3, the incidence of hypertension and tachycardia in the recovery period decreased significantly, the duration of postoperative analgesia increased significantly (*p* < 0.01) (Tables 4,5), the incidence of shivering decreased, and the onset time of the sacral canal block decreased (*p* < 0.05) (Tables 5,6).

**TABLE 4:** Incidence of adverse events of circulatory system of all groups ($\bar{\text{x}}\pm \text{SD}$, *n* = 40).

Group	Hypertension (%)	Hypotension (%)	Tachycardia (%)	Bradycardia (%)
R	20	2.5	20	2.5
D1	2.5^b^	0	2.5^b^	2.5
D2	0^b^	0	0^b^	5
D3	0^b^	0	0^b^	5

Fisher’s exact tests was used, and

a *P* < 0.05 compared with Group R

b *P* < 0.01 compared with Group R

**TABLE 5:** Analgesic effect in perioperative anesthesia of all groups ($\bar{\text{x}}\pm \text{SD}$, *n* = 40).

Group	R	D1	D2	D3
The onset time of sacral canal block (min)	14.2 ± 5.1	12.5 ± 5.0^a^	12.4 ± 4.9^a^	12.5 ± 4.8^a^
The duration of analgesia (min)	235 ± 48	466 ± 44^b^	687 ± 51^b^ ^,^ ^c^	696 ± 49^b^ ^,^ ^c^

a *P* < 0.05 compared with Group R

b *P* < 0.01 compared with Group R

c *P* < 0.01 compared with Group D1

**TABLE 6:** Incidence of adverse reactions of anesthesia of all groups ($\bar{\text{x}}\pm \text{SD}$, *n* = 40).

Group	Nausea and vomiting (%)	Shivering (%)	Pruritus (%)	Excessive sedation (%)	Bradycardia (%)	Respiratory depression (%)
R	2.5	10	0	0^c^	0^c^	0
D1	0	0^a^	0	2.5^c^	0^c^	0
D2	0	0^a^	0	2.5^c^	0^c^	0
D3	0	0^a^	0	15^c^	12.5^a^	0

Fisher’s exact tests was used, and

a *P* < 0.05 compared with Group R

b *P* < 0.01 compared with Group R

c *P* < 0.05 compared with Group D3

Compared with group D1, the duration of postoperative analgesia in groups D2 and D3 increased significantly (*p* < 0.01) (Table 5).

Compared with groups D1 and D2, the incidence of excessive sedation and bradycardia increased (*p* < 0.05) in group D3 (Table 6).

## Discussion

Children are especially susceptible to anxiety about an operation, and this may affect the smooth operation of anesthesia, lead to psychological and behavioral changes after the operation (Tucker et al., 2017; Chen and Xiong, 2018), and even produce stress reactions (Aytekin et al., 2016 Feb). As a common complication of general anesthesia for children, restlessness in the recovery period can cause an inflammatory response and is accompanied by activation of the stress response and increased excitability of the sympathetic nervous system characterized by enhancement of the function of the hypothalamus-pituitary-adrenal cortex and hyperfunction of the renin-angiotensin-aldosterone system. If large amounts of cortisol, aldosterone, and other hormones are secreted into the blood circulation, which directly mediates the stress response, it can increase the blood glucose level (Zhou et al., 2019) and cause postoperative myocardial injury and cognitive dysfunction and is positively correlated with the severity of the disease and the occurrence of multiple organ dysfunction (Zhou et al., 2018).

The ideal anesthetics can control the emergence of restlessness in the recovery period and inhibit the activation of the stress response and inflammatory response to ensure a smooth postoperative recovery process. Dexmedetomidine can activate the α2 adrenergic receptor, inhibit the impulse release of sympathetic nerve cells in the anterior horn of the spinal cord, reduce the excitability of the sympathetic nerve, activate the vagus nerve-heart reflex and baroreceptor reflex, and inhibit the stress response (Li et al., 2016). It can also reduce the dosage of anesthetics and respiratory inhibition (None. Practice, 2017).

The utilization of new anesthetics and new technologies not only ensures the safety of patients but also provides a comfortable medical experience for them. For pediatric urethroplasty, improving children’s comfort has always been a challenge. Certain measures have been taken, such as effective psychological counseling before surgery and creating a child-friendly environment to reduce fear. Applying 5% lidocaine cream to the puncture site one hour before the operation can achieve a painless puncture (Huang et al., 2018). A study revealed that 2 μg kg^−1^ of dexmedetomidine administered by nasal dripping before the operation can calm the patient, prevent postoperative restlessness, and reduce the stress response (Expert Committee of Accelerated Rehabilitation Surgery in Medical Management Center of National Health and Family Planning Commission, 2018; Sun et al., 2019). This procedure was adopted in our study during preoperative preparation.

A recent related study revealed that single anesthesia did not completely block the stress response, and different anesthesia methods could be compounded or combined and supplemented by various drugs or means to inhibit the stress response of children during the operation (Zhu, 2015). Ultrasound-guided caudal blocks have been used in our hospital for many years as well as in many other hospitals to improve patient comfort (None. Practice, 2017). The optimal dose for caudal blocks in children is 1 ml kg^−1^ of 0.2–0.3% ropivacaine (Ni et al., 2015). It has a weak motor block effect, a good analgesic effect, and low nervous system and cardiovascular toxicity. In this study, an ultrasound-guided caudal block with 0.25% ropivacaine was used. However, a caudal block with ropivacaine alone has a low anesthetic effect, and the postoperative analgesia is not satisfactory. A study confirmed that intrathecal injection of dexmedetomidine does not cause spinal cord neural injury. Conversely, it inhibits inflammatory factors and has a certain protective effect on the spinal nerve, and, therefore, it is safe (Zhang, 2015). The reason may be that the antioxidative stress effect of dexmedetomidine can stabilize the nerve cell membrane, promoting degradation and inhibiting the release of excitatory neurotransmitters such as glutamate; reduce the toxicity of excitatory amino acids to nerve cells; increase the proportion of antiapoptotic protein to pro-apoptotic protein; increase the survival number of nerve cells; and inhibit mast cell degranulation, decreasing the production of inflammatory factors and playing a neuroprotective role. Therefore, intrathecal injection of dexmedetomidine has been used in anesthesia management in various surgical procedures and has proved to be safe (Deng, 2020; Liu et al., 2020).

In related foreign studies, 1–2 μg kg^−1^ of dexmedetomidine combined with local anesthetics was used for sacral canal blocks in children. This preparation achieved good curative effects and was proven to be a safe dose (Trifa et al., 2018). Therefore, in the present study, based on the prior experiment, three different doses (1, 1.5, and 2 μg kg^−1^) of dexmedetomidine mixed with 0.25% ropivacaine were used for the sacral canal block. The results revealed that in the dexmedetomidine groups, the circulatory system was more stable, the analgesic effect was better, and the incidence of adverse events was lower. In groups D1, D2, and D3, the onset time of the caudal block decreased, the duration of analgesia increased, and the analgesia at 8 and 12 h after the operation improved significantly. The results suggest that dexmedetomidine not only has an auxiliary analgesic effect but can also prolong the block time of ropivacaine. These results are consistent with the results reported in the literature (Xu et al., 2018). The reason may be that as a highly selective α2-adrenergic receptor, dexmedetomidine can be absorbed into blood circulation or diffused into cerebrospinal fluid to produce sedative and analgesic effects on the α2A receptors of central neurons. In addition, it can activate the α2B receptors of vascular smooth muscle cells to cause vasoconstriction of the microcirculation and delay drug absorption in the sacral canal, and it can block the sensitive voltage-gated sodium channels and inhibit the action potential of the nerve cell membrane to produce a local anesthetic-like effect (Li et al., 2015). A recent study revealed that the locus coeruleus of the brainstem is the target of dexmedetomidine (Peng et al., 2018). The noradrenaline dorsal bundle fibers deriving from the locus coeruleus can regulate the arousal response of the cerebral cortex. Dexmedetomidine reduces the release of noradrenaline by acting on the α2 adrenergic receptor in the presynaptic membrane of the locus coeruleus, thereby reducing the excitability of the postsynaptic membrane and inhibiting the arousal response of the brain. The application of dexmedetomidine in general anesthesia can prevent restlessness during the recovery period and ensure a smooth recovery process (Jin et al., 2019).

It is worth noting that although dexmedetomidine has shown good efficacy, there is contradictory evidence regarding the effect of dexmedetomidine on myelination in animals, which is a warning in clinical application (Brummett et al., 2008; Konakci et al., 2008; Lirk and Brummett, 2019; Wang et al., 2019).

There are some limitations in this study. Firstly, the use of intranasal dexmedetomidine could have some impact on the results, because intranasal dexmedetomidine could have same or even stronger systemic and neuraxial anesthesia prolonging effect alone compared to sacral administration. Secondly, intranasal administration has a great influence on drug absorption, such as the degree of cleanliness and dryness of nasal cavity, the speed of infusion operation, the total drug loss caused by crying and body movement due to refusal of child, etc. Thirdly, the sample size of this study is small limited by the number of surgical cases, and large sample study is needed in the follow-up.

## Conclusion

Sacral canal application of 1.5 μg kg^−1^ of dexmedetomidine mixed with 0.25% ropivacaine in pediatric urethroplasty for hypospadias can accelerate the onset of the caudal block, effectively reduce the stress and inflammatory responses, stabilize the circulation, reduce the side effects of anesthesia, and increase the postoperative analgesia time.

## Data Availability

The original contributions presented in the study are included in the article/Supplementary Material, further inquiries can be directed to the corresponding author.
